# Neurodevelopmental differences in child and adult number processing: An fMRI-based validation of the triple code model

**DOI:** 10.1016/j.dcn.2021.100933

**Published:** 2021-02-05

**Authors:** Mikael Skagenholt, Kenny Skagerlund, Ulf Träff

**Affiliations:** a Department of Behavioral Sciences and Learning, Linköping University, Linköping, Sweden; b Department of Management and Engineering, JEDI-Lab, Linköping University, Linköping, Sweden; c Center for Social and Affective Neuroscience (CSAN), Linköping University, Linköping, Sweden

**Keywords:** Numerical cognition, Development, Children, Adults, fMRI

## Abstract

The triple code model of numerical cognition (TCM) details the neurocognitive mechanisms associated with perceiving and manipulating numerical information in exact symbolic (Arabic digits and number words) and approximate nonsymbolic numerical magnitude (e.g., dot arrays) representation codes. The current study provides a first empirical fMRI-based investigation into neurodevelopmental differences in 30 healthy children’s and 44 healthy adults’ recruitment of neural correlates associated with the Arabic digit, number word, and nonsymbolic magnitude codes. Differences between the two groups were found in cingulate regions commonly associated with domain-general aspects of cognitive control, as opposed to neural correlates of number processing per se. A primary developmental difference was identified in verbal number discrimination, where only adults recruited left-lateralized perisylvian language areas in accordance with the TCM. We therefore call for a revision of the verbal code and a formulation of separate child and adult-specific neurocognitive mechanisms associated with the discrimination of number words. Although further research is necessary, results indicate that numerical discrimination abilities in middle-school-aged children operate close to adult-level maturity. Neurodevelopmental differences may be more apparent in younger children, or on the level of functional network dynamics as opposed to a shift in recruited neural substrates.

## Highlights

• Overlapping neural responses to number discrimination tasks in adults and children.
• Ontogenetic maturation of language regions may constrain verbal number processing.
• Neural correlates of effortful number discrimination were similar across age groups.
• Prefrontal activity attributed to children’s number processing was not replicated.
• Results indicative of mature number discrimination abilities around 11 years of age.

## 1 Introduction

Symbolically represented numbers and approximate representations of quantity are essential features of daily life, scaffolding abilities such as selecting the shortest queue at the supermarket, comparing the price of two products, or producing the correct amount for payment as uttered by the clerk. Numerical discrimination tasks have demonstrated similar developmental ratio effects for both symbolic and nonsymbolic representations of number (e.g., Dehaene, 2011), such that developmental maturity affords faster and more accurate discrimination of increasingly smaller numerical ratios. Newborn infants prove unable to discriminate numerical dot arrays with ratios smaller than 1:2, but quickly develop the capacity to discriminate a ratio of 2:3 at approximately 10 months of age (e.g., Xu and Spelke, 2000). Around five years of age, similar ratio effects emerge for symbolically represented numbers (e.g., Arabic digits or number words), indicating a possible bootstrapping of number symbols onto the nonverbal approximate number system (ANS; cf. Nieder and Dehaene, 2009; Odic and Starr, 2018). An alternative account holds that symbolic number representations are separately acquired by comparison with other numerical symbols, due to inconsistent empirical evidence for overlapping performance across symbolic and nonsymbolic number codes in behavioral tests, brain imaging data, and unequal influence on mathematics achievement (e.g., Reynvoet and Sasanguie, 2016). In accordance with the bootstrapping account, the triple code model of numerical cognition (TCM; Dehaene, 1992; Dehaene and Cohen, 1995) argues for distinct but overlapping neurocognitive mechanisms recruited for the three primary representational domains of number: symbolic visual processing (e.g., “2”), symbolic verbal-auditory processing (e.g., “two”), and nonsymbolic approximate magnitude processing as supported by the ANS (e.g., “••”). Similar *distance* and *size effects* across all three formats, where reaction times increase and accuracy decreases during numerical discrimination trials featuring small numerical distances (e.g. 2 versus 3) or for larger numerosities (e.g. 8 versus 9), indicate that the formats may share a common representational basis (cf. Moyer and Landauer, 1967). While previous research has investigated and compared the neurocognitive mechanisms of number processing in up to two numerical codes, no study has yet attempted to compare the recruitment of neural correlates associated with the entire TCM in children and adults. The purpose of this study was to administer tasks related to each of the three codes, allowing for a direct empirical comparison of similarities and differences in number processing over the course of typical development. In line with this goal, our ambition was to validate previous meta-analytic research detailing the neural correlates of number processing in children (Arsalidou et al., 2018) and adults (Arsalidou and Taylor, 2011).

Approximate number system acuity (i.e., numerical discriminability) measured in infancy is a strong predictor of later math achievement (e.g., Starr et al., 2013). Park et al. (2016) demonstrated that practicing approximate arithmetic, where arrays of dots are added with subsequently presented arrays, improves symbol-based arithmetic performance in both college students and preschoolers. Practicing symbolic arithmetic has not conversely been observed to increase ANS acuity (Lindskog et al., 2016), suggesting the ANS as a primary representational system onto which symbolic numbers are mapped (cf. De Smedt et al., 2013). The TCM holds that the ANS and its primary neural correlate, the bilateral intraparietal sulcus (IPS), is commonly recruited in number discrimination tasks across all representational domains (e.g., Dehaene et al., 2003). The model additionally predicts distinct neural correlates specific to each numerical representational code. When processing Arabic digits, visual input is categorized as numerical information within the so-called *visual number form area* (VNFA) in the ventral visual stream (e.g., Grotheer et al., 2016; Yeo et al., 2017; Skagenholt et al., 2018). The left angular gyrus (AG) has been presented as an alternative neural correlate of the VNFA (e.g., Price and Ansari, 2011), as well as an upstream processing region associated with numerical and arithmetic fact retrieval (e.g., Grabner et al., 2011). For verbal representations of number, the TCM predicts increased reliance on language processing areas in the left perisylvian network (cf. Schmithorst and Douglas Brown, 2004): the inferior frontal gyrus (IFG), supramarginal gyrus (SMG), angular gyrus (AG) as well as middle and superior temporal gyri (MTG, STG).

A meta-analysis of brain areas associated with number processing (Arsalidou et al., 2018) identified a total of 32 peer-reviewed journal articles featuring participants below the age of fourteen, where 17 articles focused on number discrimination tasks as opposed to calculation tasks. No study included all three numerical codes in the same fMRI paradigm, although a number of studies in both children and adults have previously targeted up to two codes (e.g., Pinel et al., 2001; Cantlon et al., 2006; Mussolin et al., 2010). A study by Peters et al. (2016) arguably comes closest to examining the entire TCM in a sample of children, albeit with the use of calculation tasks where participants were instructed to subtract the presented number pairs. It is therefore important to extend previous research of fundamental neural substrates associated with symbolic and nonsymbolic numerical magnitude processing, in children below the age of fourteen, by including tasks relevant to all three codes in one experimental paradigm.

We hypothesized that a conjunction analysis of all three numerical formats would exhibit activity patterns largely in line with the meta-analyses previously conducted for adults (Arsalidou and Taylor, 2011) and children (Arsalidou et al., 2018). We expected participants to primarily share common neurocognitive substrates in the bilateral IPS, middle frontal gyrus (MFG), insula, right IFG, and cingulate gyrus.

At the task-level, we expected to find overlapping activity primarily in the bilateral IPS, but otherwise unique neural correlates associated with the respective numerical codes. For Arabic digit comparison, performed by adults, we expected the involvement of the left anterior cingulate cortex (ACC), IFG, STG, and middle frontal gyrus (MFG); and the right MTG, superior frontal gyrus (SFG), and IPS/AG. For verbal number comparison, we primarily expected to find activity in the left perisylvian language network and the bilateral precuneus, insula, thalamus; left ACC, hippocampus; and right MTG, caudate nucleus, IPS/AG, and SFG. Finally, for nonsymbolic magnitude comparison, we expected the involvement of early visual-stream retinotopic maps (primarily areas V2 and V3; Fornaciai et al., 2017); right IPS, SFG, IFG, caudate nucleus, and SMA; and left ACC and IFG (cf. Skagenholt et al., 2018).

Since no previous study has investigated the neural correlates associated with all three codes in the TCM for children, our hypotheses were based primarily on the meta-analysis performed by Arsalidou et al. (2018) and a selection of studies presented by the authors. Berteletti et al. (2014) performed a numerosity judgment localizer task featuring nonsymbolic dot arrays in 8–13-year-old children, finding common activation in the right SPL/IPS in line with the mapping hypothesis. In a 2-back working memory task, Libertus et al. (2009) found that eight-year-old children demonstrated unique activity to symbolic Arabic digits in the right IFG, pre- and postcentral gyri. An adult group additionally recruited the bilateral IPS (cf. Ansari et al., 2005). A potential confound is the fact that a working memory task was performed, possibly interfering with numerical magnitude mapping. Finally, Park et al. (2014) performed a functional connectivity analysis in children aged 4–6, targeting symbolic digit and nonsymbolic (dot array) number discrimination. Of primary interest is the connectivity pattern elicited between the right SPL/IPS and the left SMG as well as right precentral gyrus, possibly indicative of symbol-to-magnitude mapping. Similar patterns have been found to predict arithmetic ability in adults (Skagerlund et al., 2019). We therefore expect that symbolic number processing tasks place larger demands on the left SMG and right precentral gyrus than previously accounted for in children (Arsalidou et al., 2018).

The results of this study may provide a reference for future studies of typical and deficient numerical cognition, constituting a template of neurocognitive substrates recruited by typically developing children and young adults.

## 2 Methods

### 2.1 Participants

Thirty (*N* = 30) right-handed elementary school-aged children (ages 10–12, *Mean age* = 11.35, *SD* = 0.52, 11 girls and 19 boys) and forty-four (*N* = 44) right-handed young adult university students (ages 20–29, *Mean age* = 23.69, *SD* = 2.63, 24 female and 20 male) participated in the studies. Children performed up to an hour of mock-scanner practice. No participants had any self-reported or prior clinical documentation of mathematical difficulties, neurological illnesses, or other health issues. All participants had normal or corrected-to-normal vision. Both studies were approved by the Regional Ethical Review Board in Linköping, Sweden (adult study approval reference: 2017/103-31; child study approval reference: 2018/513-32). Adult participants were paid approximately $60, whereas participating families in the child study were not paid. Written informed consent was obtained from participating adults, and from children’s legal guardian, prior to participation. Children were asked for verbal consent.

### 2.2 Behavioral tasks

Behavioral tasks assessing reading comprehension, arithmetic fluency, and non-verbal intelligence were administered to child participants. Refer to the Supplementary Materials for further details.

### 2.3 Neuroimaging tasks

**Fig. 1:**
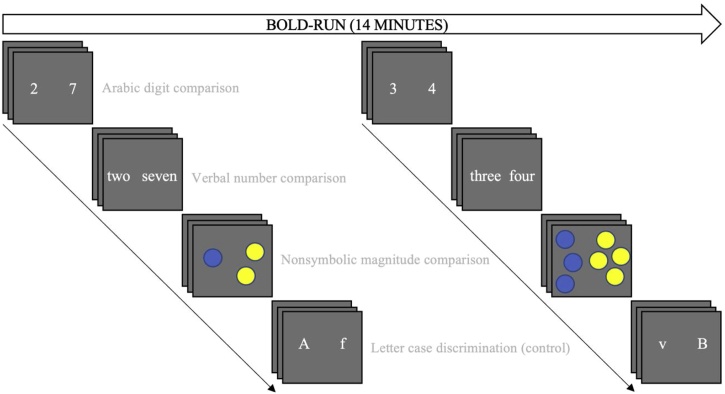
Overview of experimental trials for one BOLD run. Far-distance trials are followed by near-distance trials in a fixed order, as illustrated.

Three experimental tasks and one control task were analyzed. Each experimental task targeted one of the three codes represented in the TCM. All tasks were preceded by a fixation cross, lasting 500 ms, followed by stimulus presentation lasting 2000 ms and a response window lasting 1500 ms. Each task consisted of 14 trials, presented twice per run (alternating between far and near-distance trials for the numerical tasks), for a total of 84 trials per task across three administered BOLD (Blood-Oxygen-Level-Dependent) runs (see Fig. 1). An alternating blocked design with a fixed task order was used to minimize time between recurring instances of the same task (cf. Henson, 2007). Stimuli were presented using VisuaStimDigital video goggles (Resonance Technology Inc., Northridge, CA, USA) and participants responded by pressing the index or middle finger button on a Lumina response pad (Cedrus Corporation, San Pedro, CA, USA) placed beneath their right hand, corresponding to the left and right-hand side of the screen. Participants were instructed to respond only during the response window, as indicated by a question mark. All tasks were administered using SuperLab 5 (Cedrus Corporation, San Pedro, CA, USA).

#### 2.3.1 Arabic digit comparison

Two single Arabic digits were presented, placed to the left and right side of the screen. Participants were instructed to select the numerically larger digit by pressing the response pad button corresponding to the leftward (index finger) or rightward (middle finger) position on the screen. Half of all trials featured near-distance comparisons (i.e., a numerical distance of 1; e.g., 3 vs 4) whereas the other half featured far-distance comparisons (i.e., a numerical distance of 4–5; e.g., 2 vs 7).

#### 2.3.2 Verbal number comparison

Participants were presented with two number words in the single-digit range, positioned to the left and right-hand side of the screen, and instructed to select the numerically larger number word by pressing the corresponding response pad button. Similar to the Arabic digit comparison task, two numerical distances were administered: near-distance trials (i.e., distance 1; e.g., “three” vs “four”) and far-distance trials (i.e., distances 4–5, e.g., “two” vs “seven”).

#### 2.3.3 Nonsymbolic magnitude comparison

Numerical dot array stimuli were created using Panamath (version 1.22; Halberda et al., 2008). Two arrays of dots were simultaneously presented, to the left and right on the screen. Participants were instructed to select the most numerous dot array by pressing the corresponding response pad button during the response window. For half of all trials, cumulative surface area and numerosity matched in order to control for effects of visuospatial extent cues as opposed to pure numerosity processing. Two numerosity ratios were used in order to mimic numerical distances featured in the symbolic tasks: near-distance trials were represented by a ratio of 4:3 (e.g, 12 vs 15 dots), and far-distance trials were represented by a ratio of 1:2 (e.g., 10 vs 20 dots). Each dot array featured between 8 and 26 dots in total, in order to discourage enumeration and to avoid the presentation of stimuli within the subitizing range (e.g., Trick and Pylyshyn, 1994).

#### 2.3.4 Letter case discrimination (control)

In order to control for task-irrelevant activity associated with the experimental tasks, participants were presented with a superficially similar task featuring two alphabetical (one uppercase and one lowercase) letters presented across the horizontal plane (e.g., t vs J). Participants were instructed to select the uppercase letter, using the corresponding response pad button for the left or right-hand side.

### 2.4 fMRI data acquisition

Both fMRI studies were conducted at the Center for Medical Imaging and Visualization (CMIV), Linköping University. Neuroimaging data were acquired with a Siemens Magnetom Prisma 3.0 T MRI scanner, using a twenty-channel head coil. High-resolution T1-weighted structural scans were acquired for each subject (208 slices, 0.9 mm^3^ slice thickness, TR =2300 ms, TE =2.36 ms, flip = 8°). Three BOLD-sensitive T2*-weighted ascending Echo Planar Imaging (EPI) pulse sequence runs were performed for each participant during whole-brain functional scans (48 slices, 3.0 mm^3^ slice thickness, TR =1340 ms, TE =30 ms, flip = 69°).

### 2.5 fMRI data preprocessing

Results included in this study come from preprocessing performed using fMRIPrep 1.5.0 (Esteban et al., 2018a, 2018b), based on Nipype 1.2.2 (Gorgolewski et al., 2011, 2018). Refer to the Supplementary Materials for a boilerplate methods section generated by fMRIPrep.

### 2.6 fMRI data postprocessing

Nuisance regression was performed using fMRIDeniose (Finc et al., 2019), targeting 24 head motion parameters (3 translations, 3 rotations, temporal derivatives, and quadratic terms), 8 physiological signals (white matter and CSF with temporal derivatives and quadratic terms), and spike regression (based on framewise displacement and DVARS; Power et al., 2012). Participants were excluded if their mean framewise displacement (FD) exceeded 0.5 mm and if more than 20 % of volumes across all three BOLD runs were flagged as motion spikes (based on the criteria of FD > 0.5 mm and DVARS ± 3 SD). The remaining sample consisted of 30 children and 44 adults, as described above. Remaining data was spatially smoothed with a 4 mm full-width-at-half-maximum (FWHM) kernel in SPM 12 (Wellcome Department of Cognitive Neurology, London, UK).

### 2.7 fMRI data analysis

Whole-brain general linear model (GLM) analyses were performed using SPM 12. First-level analyses were performed at an uncorrected threshold of *p* < .001, contrasting each participant’s experimental task runs against the control task (i.e., [Task > Control]) and against parametric levels (i.e., [Near > Far]) within tasks (e.g., [Arabic_Near_ > Arabic_Far_]). Both erroneous and correct trials were included in the analysis. Voxels surviving first-level analysis were included in a second-level nonparametric two-sample T-test, conducted using the Statistical Nonparametric Mapping (SnPM version 13.1.08; http://nisox.org/Software/SnPM13/) toolbox for SPM 12. Analyses were performed at a height threshold of *p* < .001 and a familywise error corrected (FWE) cluster-forming threshold of *p* < .05. Second-level analyses were variance smoothed in accordance with the smoothing kernel (i.e., 4 mm) and subject to 10,000 permutation tests. Cluster extent thresholds for each analysis were calculated as the critical suprathreshold cluster size (STCS), as implemented in SnPM.

The use of nonparametric second-level analyses was motivated by the group size imbalance (30 versus 44 subjects) together with the use of an aggressive denoising pipeline. Both factors yield imbalanced comparative analyses, particularly given that child participants are subject to more movement and thus a loss in temporal degrees of freedom. Moreover, nonparametric statistics require few assumptions and offer strong control over the type I error rate (e.g., Nichols and Holmes, 2001).

Conjunction analyses of overlapping activity in child and adult participants, across all three tasks, were performed by subjecting FWE-corrected T-maps from individual one-sample T-tests (e.g., [Arabic_Child_ > Control_Child_]) to a minimum statistic conjunction null analysis (Nichols et al., 2005) using SPM’s ImCalc (min) function. The largest STCS cluster extent found throughout one-sample T-tests was used as the minimum extent for conjunction analyses.

## 3 Results

### 3.1 Behavioral results

Response times and accuracies for each of the tasks administered during the MRI scanning session were separately analyzed using 2 (group: child, adult) × 4 (tasks: Arabic, verbal, nonsymbolic, control) Bonferroni-corrected repeated-measures analyses of variance (ANOVA). Results are summarized in Table 1.

**Table 1:** Descriptive statistics for neuroimaging tasks.

	Reaction time		Accuracy	
Condition	*M*	*SD*	%	*SD*
Adults				
Arabic digit comparison	477.50	75.15	95.36	7.64
Verbal number comparison	480.11	79.50	95.91	6.43
Nonsymbolic magnitude comparison	503.17	87.68	93.83	6.31
Letter case discrimination (control)	476.17	82.01	95.36	6.37
Children				
Arabic digit comparison	563.18	94.74	99.52	1.02
Verbal number comparison	584.02	111.20	98.68	2.51
Nonsymbolic magnitude comparison	574.44	90.64	97.38	2.61
Letter case discrimination (control)	550.87	86.93	98.14	1.31

Response time distributions showed a main effect of group, *F*(1, 29) = 27.817, *p* < .001, η^2^_p_ = .490, and task, *F*(3, 87) = 7.399, *p* < .001, η^2^_p_ = .203, but no interaction effects. Response accuracy distributions only showed a main effect of task, *F*(2.501, 72.529) = 4.586, *p* = .008, η^2^_p_ = .137. See the Supplementary Materials for post-hoc analyses of response time and accuracy.

Refer to the Supplementary Materials for an overview of children’s reading comprehension, arithmetic fluency, and non-verbal intelligence.

### 3.2 Neuroimaging results

Probabilistic cytoarchitectonic labeling of regions was performed using the SPM Anatomy Toolbox (Eickhoff et al., 2005). In the following tables, areas in parentheses correspond to the closest identified cytoarchitectonic structures. Cluster sizes (*k*) refer to voxel count.

#### 3.2.1 Conjunction analyses

**Fig. 2:**
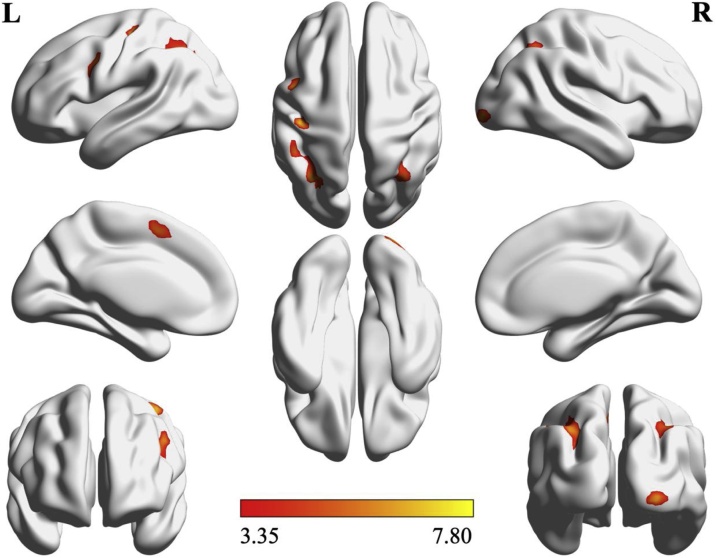
Parametric conjunction analysis of activity common to children and adults.

Conjunction analyses were performed for task–control contrasts and parametric levels within tasks. The task–control conjunction analysis indicated minimal overlap between child and adult participants in the right lingual gyrus (subdivision hOc2), featuring a cluster peak at MNI coordinates [9, -81, -6] (*k* = 85, *pseudo-T* = 5.16). This difference was primarily due to a lack of overlap between tasks in children (cf. Supplementary Table 1 and Supplementary Fig. 1), whereas adult participants demonstrated conjunction overlap in line with previous research (e.g., Skagenholt et al., 2018). The parametric conjunction analysis resulted in activity patterns within the left pre- and postcentral gyri, inferior frontal gyrus (pars Opercularis), inferior parietal lobule (IPS subdivision hIP3; SPL), cerebellum (including lobule IX, crus I lobule VI, and the cerebellar vermis), and posterior-medial frontal cortex; as well as the right inferior occipital gyrus, right inferior parietal lobule (including IPS subdivision hIP1, angular gyrus; SPL), and cerebellum (lobules VIIb and VI). See Fig. 2 and Table 2 for an overview of parametric conjunction results.

**Table 2:** Parametric conjunction (children and adults): TCM activity patterns (FWE < .05, k ≥ 49).

Anatomical region	MNI	k	*T_Pseudo_*	*p*
L Precentral gyrus	−45, 0, 36	127	7.75	< .001
L Inferior frontal gyrus (p. Oper.)	−39, 3, 30		6.50	< .001
L Postcentral gyrus	−42, -24, 57	94	7.00	< .001
L Precentral gyrus	−39, -18, 69		4.79	< .001
L Inferior parietal lobule (hIP3)	−30, -66, 45	407	6.61	< .001
L Inferior parietal lobule (BA 7A)	−36, -63, 51		6.18	< .001
L Angular gyrus (hIP3)	−30, -54, 42		5.57	< .001
R Inferior occipital gyrus (hOc1)	27, -99, -6	51	6.43	< .001
L Cerebellum (lobule IX Verm.)	−3, -54, -36	86	5.86	< .001
Cerebellar vermis (lobule I IV)	0, -51, -18		4.90	< .001
Cerebellar vermis (lobule VIIa)	0, -63, -30		4.85	< .001
L Posterior-medial frontal cortex	−9, 6, 54	72	5.45	< .001
R Inferior parietal lobule (hIP1)	27, -54, 45	143	5.43	< .001
R Angular gyrus	36, -63, 45		5.36	< .001
R Angular gyrus (BA 7A)	36, -69, 54		4.94	< .001
R Cerebellum (lobule VIIb)	12, -75, -42	110	5.41	< .001
R Cerebellar vermis (lobule VI)	6, -75, -24		5.12	< .001
L Cerebellum (crus I lobule VI)	−6, -75, -27		4.96	< .001
R Cerebellum (VIII lobule VIIb)	33, -66, -51	49	5.32	< .001
R Cerebellum (lobule VIIa crusII)	33, -78, -48		3.38	.001

Coordinates indicate peak-level activation. Rows with associated cluster sizes indicate clusters (FWE cluster-corrected), remaining regions indicate local peaks (FWE voxel-level correction).

k denotes cluster size in voxels.

#### 3.2.2 Arabic digit comparison

**Fig. 3:**
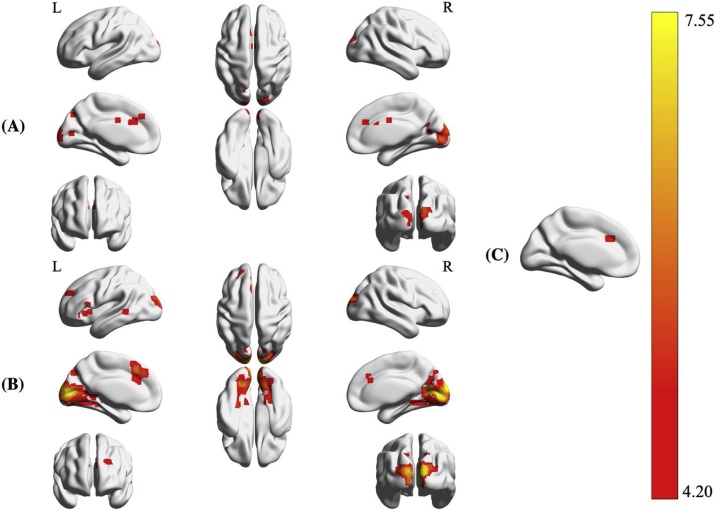
Number code-specific activity patterns unique to adults. A: [Arabic > Control] contrast. B: [Verbal > Control] contrast. C: [Nonsymbolic > Control] contrast.

A two-sample T-test indicated no suprathreshold activity unique to children’s Arabic digit comparison. Adults demonstrated unique activity in the right calcarine gyrus, cuneus, and middle cingulate cortex, as well as the left anterior cingulate cortex and cuneus. See Table 3 and Fig. 3 for an overview. For parametric two-sample T-tests, see Supplementary Table 2. Results from analyses of main effects (one-sample T-tests) for the [Arabic > Control] contrast are available in Supplementary Tables 3 (adults) and 6 (children).

**Table 3:** Regions specific to Arabic digit comparison in adults and children (FWE < .05, k ≥ 34).

Age group	Anatomical region	MNI	k	*T_Pseudo_*	*p*
Children > Adults	*No suprathreshold clusters*	–	–	–	–
Adults > Children	R Calcarine gyrus (hOc1)	6, -87, 0	382	5.53	< .001
	R Cuneus (hOc2)	9, -99, 15		5.49	.005
	R Cuneus	21, -69, 24	61	4.86	.016
	L Anterior cingulate cortex	0, 18, 27	141	4.64	.003
	R Middle cingulate cortex	0, -3, 30	49	4.52	.025
	R Calcarine gyrus (hOc1)	15, -69, 12	39	4.34	.039
	L Cuneus	−15, -75, 39	52	4.25	.021

Coordinates indicate peak-level activation. Rows with associated cluster sizes indicate clusters (FWE cluster-corrected), remaining regions indicate local peaks (FWE voxel-level correction).

k denotes cluster size in voxels.

#### 3.2.3 Verbal number comparison

Children demonstrated no unique suprathreshold activity compared to adults. Adults’ verbal number comparison elicited unique activity in the right cuneus, bilateral calcarine gyrus, and the left superior medial-frontal gyrus, anterior cingulate cortex, inferior frontal gyrus (pars Orbitalis), insula, middle temporal gyrus, and middle frontal gyrus. See Fig. 3 and Table 4 for an overview of results. For parametric two-sample T-tests, see Supplementary Table 2. Results from analyses of main effects (one-sample T-tests) for the [Verbal > Control] contrast are available in Supplementary Tables 4 (adults) and 7 (children).

**Table 4:** Regions specific to Verbal number comparison in adults and children (FWE < .05, k ≥ 34).

Age group	Anatomical region	MNI	k	*T_Pseudo_*	*p*
Children > Adults	*No suprathreshold clusters*	–	–	–	–
Adults > Children	R Cuneus (hOc3d)	9, -99, 25	2351	7.23	< .001
	L Calcarine gyrus (hOc1)	−9, -87, 6		7.13	< .001
	R Calcarine gyrus (hOc1)	12, -84, 6		7.07	< .001
	L Superior medial-frontal gyrus	−6, 24, 42	443	5.93	< .001
	L Superior medial-frontal gyrus	−6, 33, 36		5.25	.009
	L Anterior cingulate cortex	−9, 24, 30		5.00	.025
	L Inferior frontal gyrus (p. Orb.)	−51, 21, -6	174	5.04	.002
	L Insula	−30, 24, -3		4.80	.050
	L Middle temporal gyrus	−57, -42, 0	51	4.76	.025
	L Middle frontal gyrus	−21, 51, 30	52	4.44	.024

Coordinates indicate peak-level activation. Rows with associated cluster sizes indicate clusters (FWE cluster-corrected), remaining regions indicate local peaks (FWE voxel-level correction).

k denotes cluster size in voxels.

#### 3.2.4 Nonsymbolic magnitude comparison

Children demonstrated no unique suprathreshold activity. Adults showed unique activity in a single cluster (*k* = 78) within the left anterior cingulate cortex (MNI [-6, 24, 30]; *pseudo-T* = 4.97). See Fig. 3. For parametric two-sample T-tests, see Supplementary Table 2. Results from analyses of main effects (one-sample T-tests) for the [Nonsymbolic > Control] contrast are available in Supplementary Tables 5 (adults) and 8 (children).

## 4 Discussion

Neurodevelopmental differences in child and adult number processing were evaluated by fMRI analysis, featuring three tasks targeting each of the numerical codes represented in the triple code model: Arabic digit comparison (e.g., “2 vs 4”), verbal number comparison (“two vs four”), and nonsymbolic magnitude comparison (e.g., “•• vs ••••”). This approach intended to validate previous fMRI meta-analyses of the TCM (e.g., Arsalidou and Taylor, 2011; Arsalidou et al., 2018), mitigating potential confounds due to differences in data acquisition (e.g., scanners, acquisition protocols), analytic strategies (e.g., software, pre- and postprocessing), as well as tasks and experimental design.

### 4.1 Conjunction analyses of experimental tasks

The conjunction of contrasts [Arabic > Control ∩ Verbal > Control ∩ Nonsymbolic > Control] resulted in a single cluster of suprathreshold activity in the right lingual gyrus (subdivision hOc2). Leibovich and Ansari (2017) have implicated this region as one of five clusters in a bilateral occipito-parietal network, found to be more active during numerosity comparisons than brightness comparisons, suggesting low-level visual selectivity for numerical magnitude stimuli across all three representational codes of the TCM (cf. Skagenholt et al., 2018).

While the lack of additional overlapping activity patterns in children and adults could suggest developmental functional heterogeneity in numerical discrimination tasks, it is more probable that the chosen control task manifests activity patterns similar to number discrimination. One explanation could be found in the perceptual makeup of the task, where surface area is enough to distinguish lower- from uppercase letters (e.g., a vs A). The control task may therefore be treated as a magnitude comparison task in line with the spatial dimension of Walsh’s (2003) A Theory of Magnitude. Post-hoc investigation revealed that the inverse conjunction contrast (i.e., [Control > Tasks]) across age-groups produced suprathreshold activity in the bilateral intraparietal sulcus (IPS; see Supplementary Fig. 2); suggesting that absent developmental activity differences in the IPS (e.g., Ansari et al., 2005; Matejko et al., 2019) is likely attributable to the mismatched control task.

A lack of conjunction overlap is additionally due to the fact that children show minimal consistency across the three experimental tasks (see Supplementary Table and Fig. 1). A post-hoc conjunction analysis, targeting only symbolic number discrimination tasks (i.e., [Arabic > Control ∩ Verbal > Control]) across groups demonstrated substantially more overlap, suggesting that age-related differences in the processing of nonsymbolic magnitude drives the lack of overall age-independent conjunction results (see Supplementary Tables 5, 8, and 9). The symbolic task conjunction analysis demonstrated overlap in the bilateral supramarginal gyrus (SMG), postcentral gyrus, right lingual gyrus, and left insula subdivision Id1; broadly concordant with previous meta-analytic results in adult participants (Arsalidou and Taylor, 2011). The nonsymbolic conjunction analysis indicated overlap in right cerebellar lobule VI, previously implicated in working memory tasks (Stoodley et al., 2012; Cooper et al., 2012). Future research should replicate these effects with larger sample sizes, to rule out that a lack of overlap is due to deficient statistical power.

### 4.2 Parametric conjunction analysis

The parametric conjunction analysis targeting the numerical distance effect (Moyer and Landauer, 1967) produced results broadly consistent (if left-lateralized) with proposals of a dorsal and ventral frontoparietal network interacting with the IPS during number processing tasks (e.g., Sokolowski et al., 2016; Jolles et al., 2016). The use of parametric (i.e., near-distance subtracted by far-distance trials) contrasts renders it unlikely that the results feature task-irrelevant activity, given the similarity between remaining and subtracted experimental tasks. However, this approach is also likely to subtract activity common to both near and far-distance trials for each task, which limits the explanatory power of such results for the TCM as a whole.

In line with our hypotheses and previous research (e.g., Dehaene, 1992; Dehaene and Cohen, 1995; Dehaene et al., 2003; Odic and Starr, 2018), all participants across all within-task parametric subtraction contrasts demonstrated activity in the bilateral intraparietal sulcus (IPS), further cementing the region’s importance for number processing. In the frontoparietal number network, overlap between participant groups was found in substrates of the left-lateralized dorsal network: superior and inferior parietal lobe (SPL), pre- and postcentral gyri (extending towards the supplementary motor area), and a posterior-medial frontal (pMFC) region overlapping the frontal eye fields (FEF). Consistent with the ventral network, we observed activity across tasks and groups in the left inferior frontal gyrus (IFG) and bilateral angular gyrus (AG), but not the supramarginal gyrus (cf. Jolles et al., 2016). These results extend meta-analyses by Arsalidou et al. (2011, 2018) by indicating joint activity in the bilateral cerebellum (previously only identified in adult meta-analyses), as well as the left posterior-medial frontal and inferior frontal cortices (previously found exclusively for children’s calculation tasks). Joint activity across age-groups in the bilateral cerebellum particularly suggests a greater importance of neural correlates associated with verbal working memory than proposed by previous research. Overlap between participants occurred in subdivisions VIIa and VIIb crus II of the cerebellum, aligned with the phonological loop, as well as subdivision VI which has been anatomically localized as a correlate of the central executive (Cooper et al., 2012). These mechanisms overlap in the left subdivision VI crus I, mirrored by current results.

The parametric conjunction analysis indicates an overlap in neurocognitive mechanisms employed by middle-school-aged children and adults during effortful (near-distance) numerical discrimination tasks, across all three codes of the TCM. In particular, these results show greater concordance with previous meta-analytic results describing adult-specific neural correlates of number processing (Arsalidou and Taylor, 2011). This degree of overlap could explain the relative absence of age-related differences in comparisons of each individual code, as approximately 11-year-old children may already demonstrate maturation effects approaching an adult-level developmental stage.

### 4.3 Arabic digit comparison

For the Arabic digit comparison task, we hypothesized that children would exhibit unique activity in the right IFG as well as pre- and postcentral gyri, given the hypothesis that symbolic number processing migrates from the ventral attention network towards the parietal cortex as a consequence of maturing symbol mapping capabilities in ontogeny (Ansari et al., 2005; Libertus et al., 2009). On the contrary, children demonstrated no unique suprathreshold activity compared to adults. All task-related differences were attributable to adult participants, demonstrating increased activity in the bilateral cuneus, left ACC, and right calcarine and middle cingulate cortices. Given previously described overlap between child and adult participants–particularly for the symbolic codes–we are reluctant to attribute these differences to mechanisms of number processing per se, but rather indicative of domain-general neural correlates of cognitive control (cf. Shenhav et al., 2016). Since response time distributions across tasks indicated significantly faster responses in adults compared to children, without decreased accuracy, it stands to reason that the ACC and MCC in particular contribute to the allocation of attention toward conflict resolution (e.g., when a numerically larger digit is presented to the left in conflict with the counting sequence) and task-relevant stimulus properties. Note that these results were effectively mirrored in children (with the addition of the cerebellar vermis) in a two-sample T-test targeting the numerical distance effect (see Supplementary Table 2), likely indicative of increased executive demands. Future research should investigate whether functional connectivity between the cerebellum, cingulate cortex, and right IFG (nodes in the right executive function network; Habas et al., 2009) demonstrates age and effort-dependent effects.

### 4.4 Verbal number comparison

We predicted that adult activity specific for the verbal code would encompass the perisylvian language network, as well as the bilateral insula and thalamus, left hippocampus, right MTG and caudate nucleus. Since research on the verbal code is scarce in children, we had no concrete predictions beyond the finding that functional connectivity between the left SMG and right precentral gyrus positively correlates with symbolic arithmetic ability (Park et al., 2014), suggesting that these regions would be implicated across both symbol-based tasks in children. These predictions were partially fulfilled, as adults demonstrated unique left-lateralized activity in the superior medial-frontal gyrus, ACC, IFG, insula, MTG, and MFG. Children demonstrated no unique suprathreshold activity, although the left SMG and right precentral gyrus were found jointly active across groups in the symbolic conjunction analysis. These results primarily indicate neurodevelopmental differences in the recruitment of language areas (particularly IFG, insula, and MTG) during verbal number processing. White matter tractography indicates a left-lateralized ventral network (connecting the AG, STG, and SMG to the IFG) common to both linguistic semantic classification and number processing tasks (Willmes et al., 2014). The authors argue for cross-domain overlap of semantic processing in the domains of language and number, which could tentatively be argued to become more integrated over developmental time. Language-associated white matter fiber tracts undergo developmental changes from child- to adulthood, particularly in the case of a dorsal pathway (D2; connecting temporo-parietal language regions and the IFG) argued to support complex linguistic processes (Brauer et al., 2013). It may therefore be the case that a critical neurodevelopmental stage must be passed before children can make use of a jointly integrated semantic processing system for both language and number tasks. For the TCM, these results call for further research and the potential refinement of child and adult-specific variations of the verbal code. One potential avenue is to investigate whether verbal number symbols are (1) encoded as discrete lexical categories (as opposed to being compared against the ANS; Verguts et al., 2005) particularly sensitive to input frequency (cf. Lyons and Beilock, 2018), and if (2) recruitment of language regions follows as a consequence of increased exposure to written number words over the course of development.

### 4.5 Nonsymbolic magnitude comparison

Previous research on nonsymbolic magnitude discrimination suggests neurocognitive overlap in children and adults, with developmental effects primarily observed as decreased reliance on inferior frontal regions (e.g., Cantlon et al., 2009; Wilkey and Price, 2018). Current results did not indicate such developmental differences. The ability to discriminate nonsymbolic numerical magnitude develops early in ontogeny (e.g., Xu and Spelke, 2000) and has been observed in many non-human species (e.g., Dehaene, 2011), suggesting its status as a core-cognitive capacity (e.g., Carey, 2009; Núñez, 2017). A possible interpretation of absent child-specific activity is that the nonsymbolic code, as the most basic form of numerical representation, is highly developed in preadolescence compared to the symbolic codes. Adult activity in the ACC may constitute developmental refinements of number processing (or general decision-making; cf. Shenhav et al., 2016) strategies (cf. Kersey and Cantlon, 2017), but not substantial shifts in terms of employing domain-general cognitive mechanisms such as language for the symbolic codes.

### 4.6 General discussion

The results of this study are indicative of overlapping neural correlates recruited by children and adults during number processing in the Arabic, verbal, and nonsymbolic magnitude codes associated with the TCM. Overlap between the two age-groups suggests that neurocognitive mechanisms employed by approximately eleven-year-old children are highly concordant with those observed in adults (Arsalidou and Taylor, 2011). Although previous research indicates that children generally exhibit greater right-lateralized vmPFC activity in number discrimination tasks (e.g., Ansari et al., 2005), such patterns were not evident in this participant sample. We suggest three primary candidate explanations for this absence. First, activity in the right IFG has been observed to approach null or even negative beta values around 11–12 years of age when performing Arabic number discrimination (Mussolin et al., 2013), consistent with the current participant sample. This may indicate that middle-school-aged children already demonstrate maturation effects approaching adult-level performance. Note that the high-achieving nature of this sample (see primarily Raven’s SPM scores in the Supplementary Materials) could inflate such effects compared to the general population. Second, the right IFG has been associated with the performance of incongruent trials (i.e., when visual cues conflict with numerosity; Wilkey and Price, 2018), which cannot be independently evaluated in this study due to the blocked design consisting of both congruent and incongruent trials (e.g., larger digits presented to the left; larger surface area in a less numerous dot array). Third, recent research has indicated that numerical order tasks outperform discrimination tasks as predictors of mathematical achievement from third grade and onward (Lyons et al., 2014). This period in ontogeny may therefore reflect mature numerical discrimination abilities, whereas more significant neurodevelopmental differences may be found in tasks targeting numerical order.

Finally, the results raise questions regarding the mapping of numerical symbols onto the ANS (cf. Reynvoet and Sasanguie, 2016). The lack of overlapping activity between children and adults in the nonsymbolic–but not symbolic–code favors a symbol–symbol mapping account, arguing that novel symbolic numerical representations are learned from comparison with previously learned symbolic referents. Note that this outcome may be due to the mismatched control task, as the parametric conjunction analysis indicates greater neurocognitive overlap across groups and tasks. Nevertheless, it is important to note that recent multivariate neuroimaging approaches (e.g., representational similarity analysis) have begun to indicate dissimilar neural encoding patterns between numerical formats, in contrast to the mapping hypothesis (e.g., De Smedt et al., 2013), which may be a worthwhile future avenue for neurodevelopmental comparison research (cf. Lyons et al., 2015; Lyons and Beilock, 2018; Bulthé et al., 2019).

## 5 Conclusions

This study suggests an overall concordance with proposed updates to the triple code model presented by Arsalidou and Taylor (2011), although right-lateralized neural correlates were found to be more in line with Dehaene and Cohen’s (1997) sparser conception of the model. This sample of middle-school-aged children did not differ substantially from adults in their recruitment of numerosity-specific neural correlates, but rather demonstrated a lesser engagement of cingulate regions likely associated with domain-general mechanisms of cognitive control. While previous research has found stronger patterns of prefrontal activation in children across numerical discrimination tasks, such effects were not replicated. A possible explanation for the similarity between age-groups is that approximately 11-year-old children demonstrate neurodevelopmental maturity approaching that of adults. This outcome raises questions regarding critical developmental stages for numerical and mathematical cognition in relation to age, calling for further investigation into ontogenetic timepoints where differences between adult and child-level performance are most prominently observable. Future research should explore the proposal of child and adult-specific formulations of the verbal code, emerging as the primary difference identified in this participant sample. Greater neurodevelopmental differences between age-groups may be more readily observable in functional network dynamics, opposed to significant shifts in recruited neural substrates. Finally, the role of the cerebellum should be further detailed and integrated into models of children’s numerical cognition (cf. Vandervert, 2017; Arsalidou et al., 2018).

## Data statement

Associated group-level neuroimaging and behavioral data can be found at the Open Science Foundation: https://osf.io/6wrma/?view_only=31cd71d7475d4039b71654824eec5c81.

## Declaration of Competing Interest

The authors reported no declarations of interest.
