# Supplementary material for: Neurodevelopmental differences in child and adult number processing: An fMRI-based validation of the triple code model
Source: Dev Cogn Neurosci. 2021 Feb 5;48:100933. doi: 10.1016/j.dcn.2021.100933 (PMC7890357; doi:10.1016/j.dcn.2021.100933)
Supplement: Supplementary file 1 [file mmc1.docx]

**Supplementary Materials**

Neurodevelopmental differences in child and adult number processing: An fMRI-based validation of the triple code model

https://doi.org/10.1016/j.dcn.2021.100933

**Methods: fMRI data preprocessing**

Results included in this study come from preprocessing performed using fMRIPrep 1.5.0 (Esteban et al., 2018a, 2018b), which is based on Nipype 1.2.2 (Gorgolewski et al., 2011; Gorgolewski et al., 2018).

**Anatomical data preprocessing.** The T1-weighted (T1w) image was corrected for intensity non-uniformity (INU) with N4BiasFieldCorrection (Tustison et al., 2010), distributed with ANTs 2.2.0 (Avants et al., 2008), and used as T1w-reference throughout the workflow. The T1w-reference was then skull-stripped with a Nipype implementation of the antsBrainExtraction.sh workflow (from ANTs), using OASIS30ANTs as target template. Brain tissue segmentation of cerebrospinal fluid (CSF), white-matter (WM) and gray-matter (GM) was performed on the brain-extracted T1w using fast (FSL 5.0.9; Zhang, Brady, & Smith, 2001). Brain surfaces were reconstructed using recon-all (FreeSurfer 6.0.1; Dale, Fischl, & Sereno, 1999), and the brain mask estimated previously was refined with a custom variation of the method to reconcile ANTs-derived and FreeSurfer-derived segmentations of the cortical gray-matter of Mindboggle (Klein et al. 2017). Volume-based spatial normalization to one standard space (TemplateFlow ID: MNI152NLin2009cAsym) was performed through nonlinear registration with antsRegistration (ANTs 2.2.0), using brain-extracted versions of both T1w reference and the T1w template. The following template was selected for spatial normalization: ICBM 152 Nonlinear Asymmetrical template version 2009c (Fonov et al., 2009).

**Functional data preprocessing.** For each of the 3 BOLD runs per subject (across all tasks and sessions), the following preprocessing was performed. First, a reference volume and its skull-stripped version were generated using a custom methodology of fMRIPrep. The BOLD reference was then co-registered to the T1w reference using bbregister (FreeSurfer) which implements boundary-based registration (Greve & Fischl, 2009). Co-registration was configured with six degrees of freedom. Head-motion parameters with respect to the BOLD reference (transformation matrices, and six corresponding rotation and translation parameters) were estimated before any spatiotemporal filtering using mcflirt (FSL 5.0.9; Jenkinson et al., 2002). The BOLD time-series were resampled to surfaces in *fsaverage5* space. The BOLD time-series (including slice-timing correction when applied) were resampled onto their original, native space by applying a single, composite transform to correct for head-motion and susceptibility distortions. These resampled BOLD time-series will be referred to as *preprocessed BOLD in original space*, or just *preprocessed BOLD*. The BOLD time-series were resampled into standard space, generating a preprocessed BOLD run in “MNI152NLin2009cAsym” space. First, a reference volume and its skull-stripped version were generated using a custom methodology of fMRIPrep. Several confounding time-series were calculated based on the preprocessed BOLD: framewise displacement (FD), DVARS and three region-wise global signals. FD and DVARS were calculated for each functional run, both using their implementations in Nipype (following the definitions by Power et al., 2014). The three global signals were extracted within the CSF, the WM, and the whole-brain masks. Additionally, a set of physiological regressors were extracted to allow for component-based noise correction (CompCor; Behzadi et al., 2007). Principal components were estimated after high-pass filtering the preprocessed BOLD time-series (using a discrete cosine filter with 128s cut-off) for the two CompCor variants: temporal (tCompCor) and anatomical (aCompCor). tCompCor components were then calculated from the top 5% variable voxels within a mask covering the subcortical regions. This subcortical mask was obtained by heavily eroding the brain mask, which ensures it does not include cortical GM regions. For aCompCor, components were calculated within the intersection of the aforementioned mask and the union of CSF and WM masks calculated in T1w space, after their projection to the native space of each functional run (using the inverse BOLD-to-T1w transformation). Components were also calculated separately within the WM and CSF masks. For each CompCor decomposition, the *k* components with the largest singular values were retained, such that the retained components’ time series were sufficient to explain 50 percent of variance across the nuisance mask (CSF, WM, combined, or temporal). The remaining components were dropped from consideration. The head-motion estimates calculated in the correction step were also placed within the corresponding confounds file. The confound time series derived from head motion estimates and global signals were expanded with the inclusion of temporal derivatives and quadratic terms for each (Satterthwaite et al., 2013). Frames that exceeded a threshold of 0.5 mm FD or 1.5 standardised DVARS were annotated as motion outliers. All resamplings can be performed with *a single interpolation step* by composing all the pertinent transformations (i.e. head-motion transform matrices, susceptibility distortion correction when available, and co-registrations to anatomical and output spaces). Gridded (volumetric) resamplings were performed using antsApplyTransforms (ANTs), configured with Lanczos interpolation to minimize the smoothing effects of other kernels (Lanczos, 1964). Non-gridded (surface) resamplings were performed using mri_vol2surf (FreeSurfer).

Many internal operations of fMRIPrep use Nilearn 0.5.2 (Abraham et al., 2014), mostly within the functional processing workflow. For more details regarding the pipeline, see the section corresponding to workflows in the fMRIPrep documentation (https://fmriprep.readthedocs.io/en/latest/workflows.html).

**Methods: additional behavioral variables of interest**

The following section details included behavioral tasks, administered to children, featuring reading comprehension, arithmetic fluency, and non-verbal intelligence.

**Reading comprehension.** A short fictional story, spanning approximately two A4-sized pages, was presented to participating children. Twenty instances of parentheses containing three response alternatives–in the form of single words–were evenly distributed throughout the text. Participants were instructed to read as much of the story as possible within three minutes and were asked to underline the response alternative, for each parenthesis, that they perceived to be the best fit for both the current sentence as well as the overall context of the story. A total score of 20 was awarded if the child had underlined all of the correct word-alternatives for each parenthesis in three minutes or less (cf. Malmquist, 1977; Skagerlund & Träff, 2014).

**Arithmetic fluency.** The task targeted arithmetic fact retrieval, and was further divided into three subtasks for addition, subtraction, and multiplication. Participants were presented with 81 arithmetic calculations (58 for multiplication) per operation and allowed one minute to solve as many of these as possible through retrieval from long-term memory. The tasks were completed by writing answers next to the prompted problem on a piece of paper (e.g., 1+6 = 7). The children were encouraged to attempt solving as many problems as possible and were allowed to skip problems if necessary. Scores were calculated as sums of correct answers for each arithmetic operation.

**Non-verbal intelligence.** All participating children performed subsets B, C, and D of Raven’s Standard Progressive Matrices (Raven, 1976) as a measure of non-verbal intelligence. Participants were allowed 15 minutes to solve as many trials as possible. In order to derive percentile scores for each participant, obtained scores (from sets B-D) were matched against the expected score composition (Raven, Court, & Raven, 1992) for all six sets. The closest interpolated total score (prioritizing agreement with sets C and D) was then compared against percentile points associated with each individual participant’s age bracket (British norms, 1979 nationwide standardization).

**Supplementary results**

**Post-hoc tests of behavioral response time distribution.** Four Bonferroni-corrected paired t-tests indicated that adults were on average 102.09 ms faster in the Arabic number comparison task, *t*(29) = 5.538, *p* < .001; 120.71 ms faster in the verbal number comparison task, *t*(29) = 5.346, *p* < .001; 86.21 ms faster in the nonsymbolic magnitude comparison task, *t*(29) = 4.147, *p* < .001; and 92.48 ms faster in the control task, *t*(29) = 4.494, *p* < .001. Six Bonferroni-corrected paired t-tests across both age groups indicated that participants completed the Arabic digit comparison task 19.4 ms faster than the nonsymbolic magnitude comparison task, *t*(68) = 3.664, *p* < .001; and the control task was completed 23.5 ms faster than the nonsymbolic magnitude comparison task, *t*(68) = 3.966, *p* < .001.

**Post-hoc tests of behavioral response accuracy distribution.** Six Bonferroni-corrected paired t-tests were performed in order to investigate differences across all tasks irrespective of age group. Participants correctly answered 1.5 more Arabic digit comparison trials compared to nonsymbolic magnitude comparison tasks, *t*(68) = 3.538, *p* < .001; approximately 1.46 more verbal number comparison trials compared to the nonsymbolic magnitude comparison task, *t*(68) = 4.608, *p* < .001; and 1 more control trial compared to nonsymbolic magnitude comparison, *t*(68) = 2.733, *p* = .008.

**Reading comprehension.** Children read and correctly identified an average of 11.77 response alternatives (*SD* = 3.54) in the text within three minutes, ranging from a minimum of 5 to a maximum of 20 correct responses. In order to compare performance to prior research, where an approximately age-matched group (*N* = 32; *Mean age* = 10.54, *SD* = 0.29) performed the task in four minutes (Skagerlund & Träff, 2014), scores were divided by test duration and multiplied by four (i.e., (score/3)×4). The estimated mean reading score for a four-minute trial was 15.69 (*SD* = 4.72), compared to the earlier participant group’s average of 14.94 (*SD* = 4.19). A one-sample T-test did not indicate a significant difference in reading ability between samples, *t*(29) = .869, *p* = .392.

**Arithmetic fluency.** Children correctly answered an average of 26.10 addition trials (*SD* = 7.43; range: 13–44), 30.27 subtraction trials (*SD* = 8.61; range: 18–48), and 19.10 multiplication trials (*SD* = 5.69; range: 12–35) within the scope of one minute. While normative data are unavailable for these tasks, a comparison was made with unpublished data collected within the scope of a MSc thesis (Stigenberg & Tengberg, 2018; available on request from Karolinska Institutet: https://ki.se/clintec/examensarbeten-fran-logopedprogrammet). The thesis investigated mathematical abilities in a typically developing sample of children (*N* = 54; *Mean age* = 11.63; *SD* = 0.81). An independent samples T-test indicated no significant difference between samples for subtraction scores, *t*(82) = 1.055, *p* = .295; or multiplication scores, *t*(82) = 1.890, *p* = .062. Participants of the current sample correctly answered, on average, 3.12 (*SED* = 1.55) more addition trials than the reference group, *t*(82) = 2.016, *p* = .047.

**Non-verbal intelligence.** Children correctly answered an average of 30.17 out of 36 trials (*SD* = 3.34; range: 21–35) in sets B, C, and D of Raven’s Standard Progressive Matrices (Raven, 1976). The average (interpolated) percentile was 75.33 (*SD* = 25.56), indicating a high-achieving sample of participants. In total, 90% of participants (*N* = 27) scored within the 50^th^ percentile or above, with the largest frequency of scores found in the 95^th^ percentile (36.7%, *N* = 11).

**Conjunction analysis: separate task–control conjunctions for children and adults.** In order to highlight the lack of overlap between child and adult participants’ [Tasks > Control] conjunction contrasts, the following Supplementary Table 1 and Supplementary Figure 1 illustrate the lack of overlapping activity in children.

Supplementary Table 1.

*Separate [Tasks > Control] conjunction analyses for children and adults (FWE < .05, k ≥ 41)*

| Age group | Anatomical region | MNI | Cluster size | *T_Pseudo_* | *p* |
| --- | --- | --- | --- | --- | --- |
| Adults | R Lingual gyrus (hOc1) | 6, -87, -3 | 4400 | 12.80 | < .001 |
|  | R Superior occipital gyrus (hOc4lp) | 27, -87, 24 |  | 10.08 | < .001 |
|  | L Lingual gyrus (hOc3v) | -9, -84, -12 |  | 9.84 | < .001 |
|  | R Supramarginal gyrus (PFop) | 51, -30, 27 | 1156 | 8.79 | < .001 |
|  | R Insula (Ig2) | 39, -15, -3 |  | 7.87 | < .001 |
|  | R Rolandic operculum (OP3) | 39, -15, 21 |  | 6.99 | < .001 |
|  | R Middle cingulate cortex (BA 5Ci) | 15, -36, 45 | 1461 | 8.65 | < .001 |
|  | R Precentral gyrus (BA 4p) | 39, -15, 39 |  | 7.85 | < .001 |
|  | R Middle cingulate cortex | 12, -21, 39 |  | 7.57 | < .001 |
|  | L Superior temporal gyrus (Id1) | -42, -15, -6 | 624 | 7.28 | < .001 |
|  | L Superior temporal gyrus (PFcm) | -54, -33, 15 |  | 6.58 | < .001 |
|  | L Superior temporal gyrus (TE 3) | -60, -3, 6 |  | 6.57 | < .001 |
|  | L Postcentral gyrus (BA 4p) | -42, -18, 39 | 65 | 6.09 | < .001 |
|  | L Postcentral gyrus (BA 4a) | -51, -15, 45 |  | 3.81 | < .001 |
|  | L Superior frontal gyrus | -21, 33, 36 | 56 | 5.21 | < .001 |
|  | L Middle frontal gyrus | -24, 12, 51 |  | 4.59 | < .001 |
|  | L Anterior cingulate cortex | -6, 24, 30 | 113 | 5.00 | < .001 |
|  | L Anterior cingulate cortex | 0, 27, 18 |  | 4.50 | < .001 |
|  | L Superior medial-frontal gyrus | 0, 33, 33 |  | 4.02 | < .001 |
| Children | R Lingual gyrus (hOc2) | 9, -81, -6 | 88 | 5.16 | < .001 |
|  | R Lingual gyrus (hOc3v) | 21, -81, -6 |  | 4.33 | < .001 |
|  | R Fusiform gyrus (hOc3v) | 21, -81, -15 |  | 3.87 | < .001 |

Coordinates indicate peak-level activation. Left-justified rows indicate clusters (FWE-corrected at the cluster level), indented rows indicate local peaks. *k* indicates cluster size in number of voxels. Areas in parentheses correspond to closest cytoarchitectonic structures identified in SPM Anatomy Toolbox.


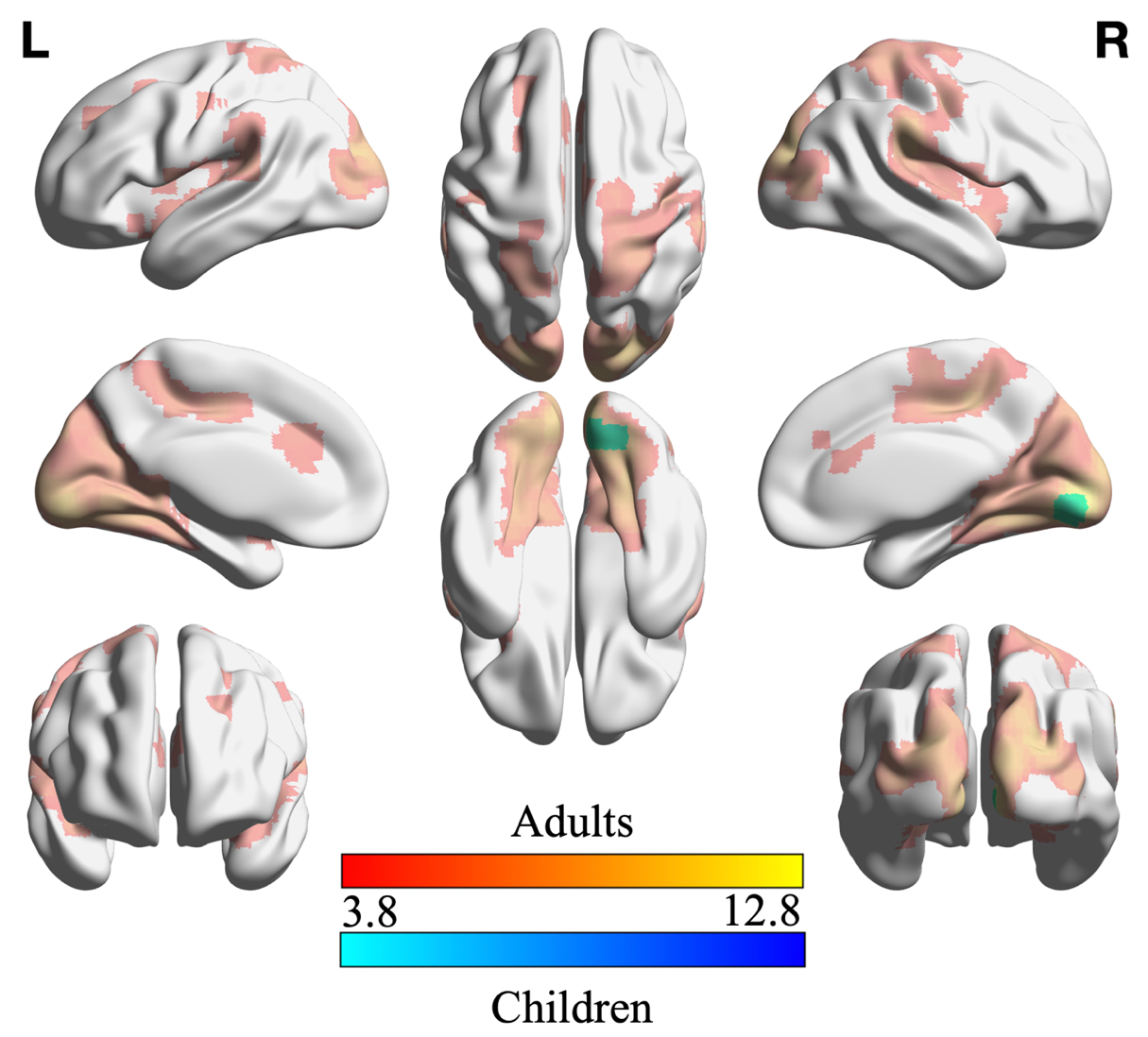


*Supplementary Figure 1.* (A) Separate [Tasks > Control] conjunction analyses for children and adults. Red: adult-specific activity. Blue: child-specific activity.

**Post-hoc analysis of [Control > Tasks] conjunction across age-groups.** In order to investigate the absence of activity in the intraparietal sulcus across [Task > Control] contrasts, an inverse conjunction analysis of [Control > Tasks] across both participant groups was performed. Results indicate that the control task, subtracted by all trials of the experimental tasks, conclusively produces suprathreshold activity in the bilateral IPS. See Supplementary Figure 2.


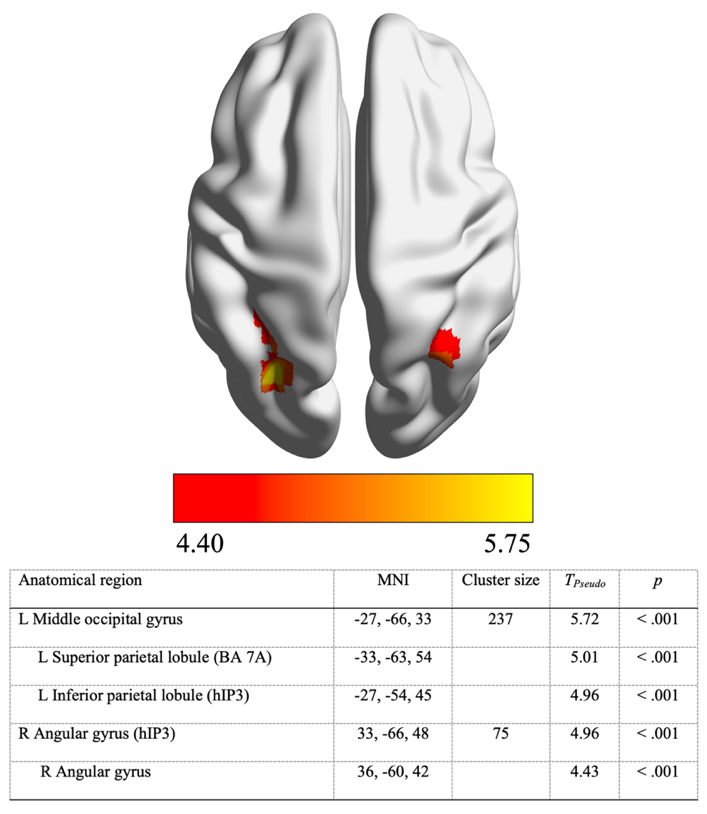


*Supplementary Figure 2.* Post-hoc analysis of conjunction [Control > Tasks] across both age-groups.

**Parametric two-sample T-tests of differences between children and adults.** In line with the numerical distance effect, activity specific to far-distance trials was subtracted from near-distance trials (i.e., the [Near>Far] contrast) for all three numerical discrimination tasks. The following Supplementary Table 2 describes suprathreshold activity unique to children and adults in the Arabic digit comparison, verbal number comparison, and nonsymbolic magnitude comparison tasks. An overview of presented results is found in Supplementary Figure 3.

Supplementary Table 2.

*Parametric task analyses (k ≥ 31, FWE-corrected peak cluster activity shown).*

| Code | Age group | Anatomical region | MNI | Cluster size | *T_Pseudo_* | *p* |
| --- | --- | --- | --- | --- | --- | --- |
| Arabic | Children > Adults | Cerebellar vermis (lobule I IV) | 0, -48, -12 | 97 | 4.72 | .006 |
|  |  | R Middle cingulate cortex | 0, -21, 27 | 48 | 4.47 | .030 |
|  |  | L Anterior cingulate cortex | -9, 24, 27 | 57 | 4.42 | .021 |
|  |  | L Precentral gyrus (BA 4a) | -39, -27, 60 | 35 | 4.23 | .050 |
|  | Adults > Children | L Inferior occipital gyrus (hOc4v) | -27, -78, -9 | 1340 | 7.00 | < .001 |
| Verbal | Children > Adults | Cerbellar vermis (lobule V) | 0, -60, -21 | 63 | 4.80 | .016 |
|  | Adults > Children | L Calcarine gyrus (hOc1) | -9, -87, 3 | 2174 | 9.20 | < .001 |
| Nonsymbolic | Children > Adults | L Middle cingulate cortex | -6, -15, 30 | 93 | 5.04 | .006 |
|  |  | R Cerebellar vermis (lobule I IV) | 3, -48, -12 | 49 | 4.69 | .022 |
|  | Adults > Children | L Lingual gyrus (hOc1) | -12, -84, 3 | 183 | 5.43 | .002 |
|  |  | L Postcentral gyrus (BA 1) | -63, -18, 39 | 78 | 5.21 | .011 |
|  |  | R Fusiform gyrus (hOc4v) | 24, -69, -6 | 283 | 5.01 | < .001 |
|  |  | R Medial temporal pole | 48, 18, -33 | 49 | 4.71 | .029 |


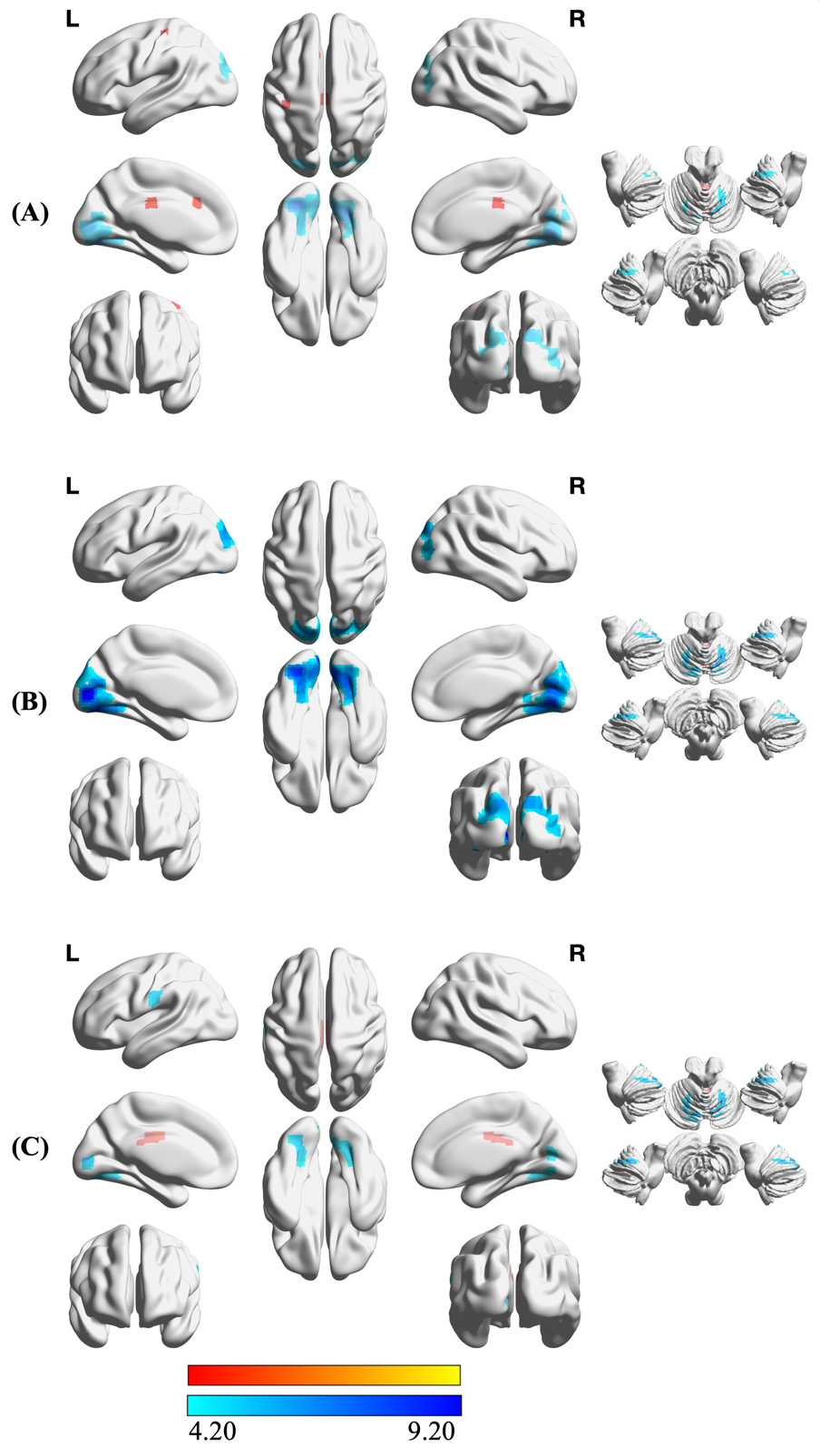


*Supplementary Figure 3.* Parametric number code-specific activity patterns in line with the distance effect ([Near > Far] contrasts). Blue clusters: adult-specific activity. Red clusters: child-specific activity. A: Arabic digit comparison. B: Verbal number comparison C: Nonsymbolic magnitude comparison.

**Main effects of [Task > Control] contrasts.** Six nonparametric one-sample T-tests (FWE-corrected at *p* < .05 at the second level) were performed in order to investigate main effects of each task associated with the TCM as subtracted by the control task. Supplementary Tables 2–7 describe main effects of the Arabic, verbal, and nonsymbolic magnitude comparison tasks for adults and children.

Supplementary Table 3.

| Anatomical region | MNI | *k* | *Pseudo-T* | *p* |
| --- | --- | --- | --- | --- |
| R Lingual gyrus (hOc1) | 6, -87, -3 | 10167 | 13.87 | < .001 |
| R Cuneus (hOc2) | 12, -99, 15 |  | 12.28 | < .001 |
| L Lingual gyrus (FG3) | -30, -45, -6 |  | 12.12 | < .001 |
| L Superior temporal gyrus (Ig2) | -42, -15, -3 | 1131 | 8.91 | < .001 |
| L Superior temporal gyrus (PFcm) | -54, -33, 15 |  | 8.07 | < .001 |
| L Superior temporal gyrus (Id1) | -42, -6, -12 |  | 7.90 | < .001 |
| L Postcentral gyrus (BA 4p) | -39, -18, 39 | 123 | 7.79 | .009 |
| R Thalamus (Parietal) | 21, -24, -6 | 54 | 7.24 | .035 |
| L Caudate nucleus | -6, 6, 9 | 49 | 7.10 | .040 |
| L Middle frontal gyrus | -24, 12, 48 | 281 | 5.79 | .003 |
| L Superior frontal gyrus | -21, 33, 36 |  | 5.54 | .004 |
| L Middle frontal gyrus | -27, 39, 21 |  | 5.36 | .009 |
| L Anterior cingulate cortex | -3, 21, 27 | 140 | 5.63 | .008 |

*Adults: Arabic>Control (one-sample T-test; k ≥* 41)

Coordinates indicate peak-level activation. Left-justified rows indicate clusters (FWE-corrected at the cluster level), indented rows indicate local peaks (FWE-corrected at the voxel level). *k* indicates cluster size in number of voxels. Areas in parentheses correspond to closest cytoarchitectonic structures identified in SPM Anatomy Toolbox.

Supplementary Table 4.

| Anatomical region | MNI | *k* | *Pseudo-T* | *p* |
| --- | --- | --- | --- | --- |
| R Lingual gyrus (hOc1) | 6, -87, -3 | 7100 | 13.29 | < .001 |
| R Middle occipital gyrus (hOc4d) | 27, -87, 21 |  | 10.15 | < .001 |
| L Lingual gyrus (hOc2) | -12, -63, -3 |  | 9.92 | < .001 |
| R Supramarginal gyrus (PFop) | 51, -30, 27 | 5273 | 10.23 | < .001 |
| R Precentral gyrus (BA 4p) | 39, -15, 39 |  | 9.68 | < .001 |
| R Middle cingulate cortex (BA 5Ci) | 15, -33, 42 |  | 9.13 | < .001 |
| L Postcentral gyrus (BA 4p) | -42, -18, 39 | 98 | 7.81 | .013 |
| L Caudate nucleus | -6, 6, 9 | 103 | 6.71 | .013 |
| L Caudate nucleus | -12, 0, 15 |  | 5.94 | .001 |
| R Superior frontal gyrus | 24, 42, 36 | 119 | 5.27 | .010 |
| R Caudate nucleus | 12, 6, 12 | 53 | 5.04 | .033 |

*Adults: Verbal>Control (one-sample T-test; k ≥* 39)

Coordinates indicate peak-level activation. Left-justified rows indicate clusters (FWE-corrected at the cluster level), indented rows indicate local peaks (FWE-corrected at the voxel level). *k* indicates cluster size in number of voxels. Areas in parentheses correspond to closest cytoarchitectonic structures identified in SPM Anatomy Toolbox.

Supplementary Table 5.

| Anatomical region | MNI | *k* | *Pseudo-T* | *p* |
| --- | --- | --- | --- | --- |
| R Lingual gyrus (hOc1) | 6, -87, -3 | 4801 | 12.80 | < .001 |
| R Cuneus (hOc2) | 12, -99, 15 |  | 11.41 | < .001 |
| R Superior occipital gyrus (hOc4d) | 24, -87, 21 |  | 10.83 | < .001 |
| R Supramarginal gyrus (PFop) | 51, -30, 27 | 1386 | 8.79 | .001 |
| R Insula (Ig2) | 39, -15, -3 |  | 7.87 | < .001 |
| R Rolandic operculum (OP3) | 39, -15, 21 |  | 7.00 | < .001 |
| R Middle cingulate cortex (BA 5Ci) | 15, -36, 45 | 1976 | 8.65 | < .001 |
| R Precentral gyrus (BA 4p) | 39, -15, 39 |  | 7.85 | < .001 |
| R Middle cingulate cortex | 12, -21, 39 |  | 7.57 | < .001 |
| L Superior temporal gyrus (Id1) | -42, -15, -6 | 733 | 7.28 | .001 |
| L Superior temporal gyrus (TE 3) | -60, -3, 6 |  | 6.64 | < .001 |
| L Superior temporal gyrus (PFcm) | -54, -33, 15 |  | 6.58 | < .001 |
| Thalamus | 0, -21, -3 | 73 | 6.34 | .019 |
| L Postcentral gyrus (BA 4p) | -42, -18, 39 | 67 | 6.09 | .021 |
| L Superior frontal gyrus | -21, 33, 36 | 56 | 5.21 | .028 |

*Adults: Nonsymbolic>Control (one-sample T-test; k ≥* 38)

Coordinates indicate peak-level activation. Left-justified rows indicate clusters (FWE-corrected at the cluster level), indented rows indicate local peaks (FWE-corrected at the voxel level). *k* indicates cluster size in number of voxels. Areas in parentheses correspond to closest cytoarchitectonic structures identified in SPM Anatomy Toolbox.

Supplementary Table 6.

| Anatomical region | MNI | *k* | *Pseudo-T* | *p* |
| --- | --- | --- | --- | --- |
| R Lingual gyrus (hOc2) | 9, -81, -6 | 2515 | 8.33 | < .001 |
| R Middle occipital gyrus (hOc4la) | 39, -78, 3 |  | 7.76 | < .001 |
| R Lingual gyrus (hOc3v) | 21, -69, -12 |  | 7.55 | < .001 |
| R Postcentral gyrus (BA 2) | 33, -39, 60 | 1268 | 7.56 | < .001 |
| R Supramarginal gyrus (PFop) | 51, -30, 24 |  | 6.65 | .001 |
| R Superior temporal gyrus (PFcm) | 60, -27, 18 |  | 6.37 | .002 |
| L Superior temporal gyrus (PF/PFcm) | -60, -33, 21 | 397 | 6.38 | .001 |
| L Postcentral gyrus (BA 4p) | -42, -18, 36 |  | 5.91 | .004 |
| L Postcentral gyrus (BA 1) | -63, -18, 39 |  | 5.38 | .016 |
| L Postcentral gyrus (BA 2) | -27, -39, 66 | 170 | 5.77 | .006 |
| L Postcentral gyrus (BA 2) | -30, -39, 54 |  | 5.74 | .006 |
| L Amygdala | -30, 3, -18 | 85 | 5.32 | .017 |
| R Middle temporal gyrus | 54, -3, -21 | 86 | 5.03 | .017 |
| R Paracentral lobule (BA 5Ci) | 12, -36, 51 | 64 | 4.38 | .031 |

*Children: Arabic>Control (one-sample T-test; k ≥* 47)

Coordinates indicate peak-level activation. Left-justified rows indicate clusters (FWE-corrected at the cluster level), indented rows indicate local peaks (FWE-corrected at the voxel level). *k* indicates cluster size in number of voxels. Areas in parentheses correspond to closest cytoarchitectonic structures identified in SPM Anatomy Toolbox.

Supplementary Table 7.

*Children: Verbal>Control (one-sample T-test; k ≥* 44)

| Anatomical region | MNI | *k* | *Pseudo-T* | *p* |
| --- | --- | --- | --- | --- |
| R Superior temporal gyrus (PFcm) | 60, -27, 18 | 770 | 6.34 | < .001 |
| R Supramarginal gyrus (PFop) | 51, -30, 24 |  | 6.09 | .001 |
| R Precentral gyrus (BA 3b) | 51, -12, 42 |  | 5.81 | .004 |
| L Supramarginal gyrus (PF) | -60, -33, 24 | 448 | 6.15 | < .001 |
| L Supramarginal gyrus (BA 4p) | -51, -12, 33 |  | 5.30 | .020 |
| L Postcentral gyrus (BA 4p) | -39, -18, 39 |  | 5.21 | .025 |
| R Postcentral gyrus (BA 2) | 30, -42, 63 | 240 | 5.76 | .001 |
| R Postcentral gyrus (BA 2) | 27, -39, 54 |  | 5.59 | .009 |
| R Superior parietal lobule (BA 5L) | 21, -54, 63 |  | 5.31 | .019 |
| R Temporal pole | 39, 6, -18 | 45 | 5.65 | .050 |
| R Lingual gyrus (hOc2) | 9, -81, -6 | 90 | 5.16 | .015 |
| L Postcentral gyrus (BA 2) | -27, -39, 66 | 121 | 5.13 | .007 |
| L Precuneus (BA 5L) | -15, -54, 75 |  | 5.07 | .038 |

Coordinates indicate peak-level activation. Left-justified rows indicate clusters (FWE-corrected at the cluster level), indented rows indicate local peaks (FWE-corrected at the voxel level). *k* indicates cluster size in number of voxels. Areas in parentheses correspond to closest cytoarchitectonic structures identified in SPM Anatomy Toolbox.

Supplementary Table 8.

| Anatomical region | MNI | *k* | *Pseudo-T* | *p* |
| --- | --- | --- | --- | --- |
| R Cerebellum (VI) | 21, -75, -15 | 2654 | 9.66 | < .001 |
| R Lingual gyrus (hOc2) | 9, -78, -6 |  | 9.08 | < .001 |
| L Middle occipital gyrus (hOc4d) | -18, -87, 18 |  | 8.19 | < .001 |

*Children: Nonsymbolic>Control (one-sample T-test; k ≥* 40)

Coordinates indicate peak-level activation. Left-justified rows indicate clusters (FWE-corrected at the cluster level), indented rows indicate local peaks (FWE-corrected at the voxel level). *k* indicates cluster size in number of voxels. Areas in parentheses correspond to closest cytoarchitectonic structures identified in SPM Anatomy Toolbox.

**Separate symbolic and nonsymbolic conjunction analyses.** Post-hoc conjunction analyses, separately targeting symbolic (i.e., Arabic and verbal) and nonsymbolic numerical magnitude discrimination tasks subtracted by the control task, indicated greater overlap for symbolic than nonsymbolic tasks across age-groups. See Supplementary Table 9.

Supplementary Table 9.

*Separate symbolic and nonsymbolic task conjunction analyses (all participants; k ≥* 40*)*

| Conjunction | Anatomical region | MNI | Cluster size | *T_Pseudo_* | *p* |
| --- | --- | --- | --- | --- | --- |
| Symbolic | R Superior temporal gyrus (PFcm) | 60, -27, 18 | 601 | 6.34 | < .001 |
|  | L Supramarginal gyrus (PF) | -60, -33, 24 | 132 | 6.15 | < .001 |
|  | R Postcentral gyrus (BA 2) | 30, -42, 63 | 223 | 5.76 | < .001 |
|  | R Lingual gyrus (hOc2) | 9, -81, -6 | 86 | 5.16 | < .001 |
|  | L Postcentral gyrus (BA 2) | -27, -39, 66 | 90 | 5.04 | < .001 |
|  | L Superior temporal gyrus (Id1) | -42, -15, -6 | 51 | 4.47 | < .001 |
| Nonsymbolic | R Cerebellum (lobule VI) | 21, -75, -15 | 2388 | 9.66 | < .001 |

Coordinates indicate peak-level activation. FWE-corrected cluster peaks shown. *k* indicates cluster size in number of voxels. Areas in parentheses correspond to closest cytoarchitectonic structures identified in SPM Anatomy Toolbox.

Supplementary References

Abraham, A., Pedregosa, F., Eickenberg, M., Gervais, P., Mueller, A., Kossaifi, J., … Varoquaux, G. (2014). Machine learning for neuroimaging with scikit-learn. *Frontiers in Neuroinformatics*, *8*(14), https://doi.org/10.3389/fninf.2014.00014.

Avants, B., Epstein, C., Grossman, M., & Gee, J. (2008). Symmetric diffeomorphic image registration with cross-correlation: Evaluating automated labeling of elderly and neurodegenerative brain. Medical Image Analysis, 12(1), 26–41.

Behzadi, Y., Restom, K., Liau, J., & Liu, T. T. (2007). A component based noise correction method (CompCor) for BOLD and perfusion based fMRI. NeuroImage, 37(1), 90–101.

Dale, A. M., Fischl, B., & Sereno, M. I. (1999). Cortical Surface-Based Analysis: I. Segmentation and Surface Reconstruction. NeuroImage, 9(2), 179–94.

Fonov, V., Evans, A., Mckinstry, R., Almli, C., & Collins, D. (2009). Unbiased nonlinear average age-appropriate brain templates from birth to adulthood. NeuroImage, 47.

Greve, D. N., & Fischl, B. (2009). Accurate and robust brain image alignment using boundary-based registration. NeuroImage, 48(1), 63–72.

Jenkinson, M., Bannister, P., Brady, M., & Smith, S. (2002). Improved Optimization for the Robust and Accurate Linear Registration and Motion Correction of Brain Images. NeuroImage, 17(2), 825–841.

Klein, A., Ghosh, S. S., Bao, F. S., Giard, J., Häme, Y., Stavsky, E., … Keshavan, A. (2016). Mindboggling morphometry of human brains. PLOS Computational Biology, 13(2), e1005350.

Lanczos, C. (1964). Evaluation of Noisy Data. Journal of the Society for Industrial and Applied Mathematics Series B Numerical Analysis, 1(1), 76–85.

Malmquist, E. (1977). Reading and Writing Difficulties in Children. Analysis and Treatment. Lund: Gleerups.

Power, J. D., Mitra, A., Laumann, T. O., Snyder, A. Z., Schlaggar, B. L., & Petersen, S. E. (2014). Methods to detect, characterize, and remove motion artifact in resting state fMRI. NeuroImage, 84, 320–341.

Raven, J. C. (1976). Standard Progressive Matrices. Oxford: Oxford Psychologists Press.

Raven, J. C., Court, J. H., & Raven, J. (1992). Standard Progressive Matrices. Oxford: Oxford Psychologists Press.

Satterthwaite, T. D., Elliott, M. A., Gerraty, R. T., Ruparel, K., Loughead, J., Calkins, M. E., … Wolf, D. H. (2013). An improved framework for confound regression and filtering for control of motion artifact in the preprocessing of resting-state functional connectivity data. NeuroImage, 64, 240–256.

Skagerlund, K., & Träff, U. (2014). Development of magnitude processing in children with developmental dyscalculia: space, time, and number. Frontiers in Psychology, 5(675), 1–15.

Tustison, N. J., Avants, B. B., Cook, P. A., Zheng, Y., Egan, A., Yushkevich, P. A., & Gee, J. C. (2010). N4ITK: Improved N3 Bias Correction. IEEE Transactions on Medical Imaging, 29(6), 1310–1320.

Zhang, Y., Brady, M., & Smith, S. (2001). Segmentation of brain MR images through a hidden Markov random field model and the expectation-maximization algorithm. IEEE Transactions on Medical Imaging, 20(1), 45–57.
